# Cases of Successful Version, with Living Children, after Failure of the Forceps

**Published:** 1871-06

**Authors:** J. J. Phillips

**Affiliations:** London, Assistant Obstetric Physician to Guy’s Hospital, Assistant Physician to the Children’s Hospital, and Physician to the Royal Maternity Charity


*Cases of Successful Version, with Living Children, after Failure of the Forceps.*—By J. J. Phillips, M.D., London, Assistant Obstetric Physician to Guy's Hospital, Assistant Physician to the Children's Hospital, and Physician to the Royal Maternity Charity.

---

The following cases, which have occurred to me during the past year, are recorded as affording additional evidence of the superiority of version over the forceps in certain casas of coarctation of the pelvic brim.

Case 1.—On April 25th, 1870, I saw, at Islington, a patient in labor with her seventh child. Her first pregnancy terminated prematurely at the seventh month, but the infant did not long survive its birth. This was the only child born alive. To this succeeded other premature deliveries; but it was believed that the fifth child and the sixth were born at term. In her last labor she was seen by a distinguished physician, who, failing to complete the delivery by the forceps, performed version, and delivered her of a still-born male child.

The patient was short in statue, but there did not exist any apparent deformity of the spine or of the lower limbs. When I saw her, on the present occasion, she had been about fifteen hours in labor, and the os uteri had been fully dilated for some hours, but the child's head had not entered the pelvis. The pulse was of good power, and there were strong rhythmical uterine pains. The sacral promontory was easily reached by the finger. It was determined to administer chloroform, I then explored the pelvis, and found that the antero-posterior diameter at the brim, as accurately as I could measure it, was very little over three inches. I applied the long double-curved forceps, though I hardly expected to succeed with them; but no justifiable amount of traction produced any effect in bringing the head through the brim. I then seized a foot: the child rotated easily, and, as the soft parts were dilatable, the lower half of the body was soon born. The shoulders were hooked down by carrying the trunk first well forward and then backward. I anticipated considerable difficulty in extracting the head, but steady traction with one hand on the back of the neck, and the other on the legs, soon brought the head into the cavity with a slight jerk. The child—a good-sized female one—was born asphyxiated, but was resuscitated by artificial respiration, though it was about twenty minutes before she breathed vigorously. I heard subsequently that the mother and the child progressed favorably.

Case 2.—On the evening of the 3rd of July, I was called to a patient of the Royal Maternity Charity, aged forty-one, who was in labor with her sixteenth child. Of the fifteen children, seven only had been born alive. She had been delivered of the last five "by instruments," and of these four were dead. The patient had neglected repeated warnings to have premature labor induced. The midwife said she had been delivered once by my predecessor in the Charity, Dr. Barnes, and that he advised labor to be brought on, in any subsequent pregnancy, about the seventh or eighth month. I found a fat woman, complaining loudly, and urgently requesting instrumental assistance. The liquor amnii had escaped eight hours previously, and since then there had been strong labor pains. The abdomen was very pendulous, the uterus lying forward, and the child's head had very slightly entered the brim. The pulse beat 130 per minute. I endeavored to restore the uterus to its proper axis by means of a broad binder, and subsequently applied the long double-curved forceps, which locked easily. I felt confident at first that the forceps would succeed; but after using as much compressive power and firm traction for a considerable time as I deemed compatible with the life of the child, I desisted. Dr. Richards then kindly placed the patient under the influence of chloroform, and, introducing my hand, I carefully examined the brim of the pelvis. The conjugate diameter was estimated at about three inches and a half, but the fœtal head was very large and firm. I turned the child without difficulty. The head engaged in the pelvis in the left oblique diameter, its liberation was effected after meeting with much resistance. The child, a large male, though at first apparently dead, soon recovered. The mother suffered from chronic bronchitis and emphysema, and her convalescence was rather slow, as indeed it had been after previous labors.

Case 3.—Early in the morning of the 18th of February, I was summoned to a case of difficult labor, in which repeated attempts had been made to deliver by means of the long forceps. The history which I obtained was that the patient was the mother of three or four children; that each successive labor had been increasingly difficult; and that the last had terminated, with the aid of the forceps, in the birth of a dead child. The os uteri was dilated to the size of half-a-crown on the preceding morning; and at seven in the evening it was fully dilated, and the pains were frequent and very strong. Six hours later, as the fœtal head was pressed into the pelvic brim, but would not pass through it, the forceps were applied; but the attempts to bring the head into the cavity were unavailing. I turned the child slowly but easily, the patient being under the influence of chloroform. The head became again fixed at the brim for four or five minutes, but was then liberated by firm traction. Contrary to expectation, the child, after a little attntion had been paid it, began to breathe, and subsequently to cry lustily. I have no note of the estimated dimensions of the pelvis in this case ; but the obstruction was due to a jutting sacral promontory, which had produced a marked depression on the left parietal and frontal bones of the fœtal head.

*Finsbury Square, February,* 1871.

